# Multidisciplinary nephrogenetic outpatient clinic combined with diagnostic exome sequencing for improved diagnostics and treatment

**DOI:** 10.1186/2046-2530-4-S1-P53

**Published:** 2015-07-13

**Authors:** D Lugtenberg, H Arts, J Van Reeuwijk, E Cornelissen, J Deegens, J Hofstra, J Wetzels, C Gilissen, R Roepman, E Kamsteeg, E Bongers

**Affiliations:** 1 Human Genetics, Radboud University Medical Center, Nijmegen, The Netherlands; 2 Pediatric Nephrology, Radboud University Medical Center, Nijmegen, The Netherlands; 3 Nephrology, Radboud University Medical Center, Nijmegen, The Netherlands

## 

Single gene disorders are estimated to account for ~30% of children and ~10% of adult patients attending renal outpatient services. For mutation detection by exome sequencing, deep phenotyping, reverse phenotyping and family history information are important. A multidisciplinary nephrogenetic outpatient clinic for children and adult patients with (genetic) kidney diseases has been established by a team of (pediatric) nephrologists and a clinical geneticist in the Radboudumc. Clinical exome sequencing for a broad spectrum of isolated- and syndromic renal (ciliary) disorders has been developed. The approach consists of a two-tier analysis in which the first step is to screen for pathogenic variants in genes that are known to be mutated in renal diseases (170 genes) or (renal) ciliopathies (125 genes). If causative mutations are not identified in the first step, the complete exome data set can be analysed with informed consent. The first results with the renal disease gene panel in 35 unrelated patients with undiagnosed renal disease led to pathogenic mutations in the *CC2D2A, CLCN5, NPHP1 *and *UMOD *gene, detected in four cases and in six other cases likely pathogenic variants needed follow-up studies. Further analysis of the complete exome data set in 13 patients, revealed possible pathogenic mutations in two cases. While variant and copy number variation analysis in the rest of exome is expected to further increase diagnostic yield, we can already conclude that the combination of the multidisciplinary outpatient clinic with diagnostic exome sequencing provides a powerful tool for detecting causative mutations of renal disease.

